# Should dogs and cats be fed vegan diets?

**DOI:** 10.3389/fvets.2024.1430743

**Published:** 2024-08-01

**Authors:** Faraz Harsini, Andrew Knight, Benny Smith

**Affiliations:** ^1^Allied Scholars for Animal Protection, Austin, TX, United States; ^2^School of Veterinary Medicine, College of Environmental and Life Sciences, Murdoch University, Murdoch, WA, Australia; ^3^School of Environment and Science, Griffith University, Nathan, QLD, Australia; ^4^Faculty of Health and Wellbeing, University of Winchester, Winchester, United Kingdom

**Keywords:** vegan, pet, nutrition, dog, cat, veterinary, plant-based, vegetarian

Vegan pet diets have historically been controversial, as dogs and cats are biologically omnivores and carnivores respectively. However, due to the demands of consumers concerned about farmed animal welfare and environmental sustainability, increasing numbers of pet food companies are now producing vegan diets excluding any animal products. These aim to supply all nutritional needs using plant-sourced ingredients, and supplements of minerals, vitamins and amino acids, amongst others.

However, a recent study by Daina et al. (1) asserted nutritional inadequacies in vegan pet diets. The study based its conclusions on the analysis of only three specific diets—a sample insufficient to draw conclusions about the nutritional soundness of all vegan pet diets. Nutritional unsoundness is also not uncommon among nonvegan pet diets (2). Although diets in each group may be nutritionally sound or unsound, depending on the quality of diet formulation and manufacturing, systemic differences between vegan and meat-based pet foods appear minimal in this respect. In fact, a recent survey of 29 pet food manufacturers (many more than examined by Daina et al.), which examined steps taken to ensure nutritional soundness and diet quality, found that 10 plant-based pet foods had slightly higher standards overall, than 19 meat-based pet foods (3). The former were more—not less—likely to be nutritionally sound.

Furthermore, the gold standard test for nutritional adequacy is animal health and longevity. Ten studies in dogs (4–13) and three in cats (14–16) have found that vegan diets produce health outcomes as good or better than nonvegan diets. The palatability of vegan pet diets appears comparable to that of meat-based diets (17), and nutritionally-sound vegan diets for dogs and cats offer major benefits for environmental sustainability (18).

The sweeping claims made by Daina et al. concerning the nutritional unsoundness of vegan pet diets are inconsistent with the evidence in this field, and incorrect. Given the positive health outcomes for dogs and cats maintained on nutritionally-sound vegan diets, and the substantial environmental benefits such diets may offer, the use of such diets should be supported.

## Author contributions

FH: Conceptualization, Investigation, Writing – original draft, Writing – review & editing. AK: Investigation, Writing – original draft, Writing – review & editing. BS: Writing – original draft, Writing – review & editing, Investigation.
